# Mimicking the Mammalian Plasma Membrane: An Overview of Lipid Membrane Models for Biophysical Studies

**DOI:** 10.3390/biomimetics6010003

**Published:** 2020-12-31

**Authors:** Alessandra Luchini, Giuseppe Vitiello

**Affiliations:** 1 Niels Bohr Institute, University of Copenhagen, Universitetsparken 5, 2100 Copenhagen, Denmark; a.luchini@nbi.ku.dk; 2 Department of Chemical, Materials and Production Engineering, University of Naples Federico II, Piazzale Tecchio 80, 80125 Naples, Italy; 3 CSGI-Center for Colloid and Surface Science, via della Lastruccia 3, 50019 Sesto Fiorentino (Florence), Italy

**Keywords:** mammalian plasma membrane, biomimetic lipid membranes, biomimicking models, protein-membrane interactions, drug-membrane interactions

## Abstract

Cell membranes are very complex biological systems including a large variety of lipids and proteins. Therefore, they are difficult to extract and directly investigate with biophysical methods. For many decades, the characterization of simpler biomimetic lipid membranes, which contain only a few lipid species, provided important physico-chemical information on the most abundant lipid species in cell membranes. These studies described physical and chemical properties that are most likely similar to those of real cell membranes. Indeed, biomimetic lipid membranes can be easily prepared in the lab and are compatible with multiple biophysical techniques. Lipid phase transitions, the bilayer structure, the impact of cholesterol on the structure and dynamics of lipid bilayers, and the selective recognition of target lipids by proteins, peptides, and drugs are all examples of the detailed information about cell membranes obtained by the investigation of biomimetic lipid membranes. This review focuses specifically on the advances that were achieved during the last decade in the field of biomimetic lipid membranes mimicking the mammalian plasma membrane. In particular, we provide a description of the most common types of lipid membrane models used for biophysical characterization, i.e., lipid membranes in solution and on surfaces, as well as recent examples of their applications for the investigation of protein-lipid and drug-lipid interactions. Altogether, promising directions for future developments of biomimetic lipid membranes are the further implementation of natural lipid mixtures for the development of more biologically relevant lipid membranes, as well as the development of sample preparation protocols that enable the incorporation of membrane proteins in the biomimetic lipid membranes.

## 1. Introduction

**Figure 1:**
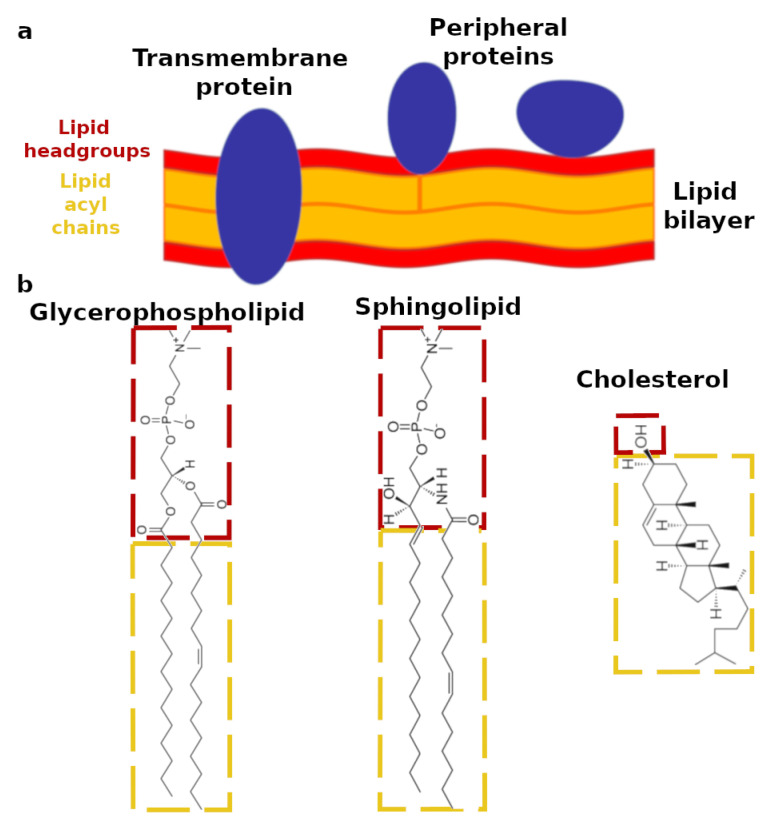
(**a**) Schematic representation of a lipid bilayer with membrane proteins. Transmembrane proteins are located across the lipid bilayer; peripheral proteins are mainly located at the bilayer surface; in some cases, they can include a fatty acid moiety in their structure, which is embedded in the lipid bilayer. The lipid bilayer is composed of two layers of lipids; the lipid headgroups are reported in red, and the lipid acyl chains are reported in yellow. (**b**) Structure of the three main classes of lipids in the plasma membrane. As an example of glycerophospholipids and sphingolipids, the chemical structures of 1-palmitoyl-2-oleoyl-sn-glycero-3-phosphocholine and *N*-palmitoleoyl-d-erythro-sphingosylphosphorylcholine are reported, respectively. The lipid hydrophilic headgroup is outlined in red, whereas the lipid hydrophobic acyl chains are outlined in yellow.

Cell membranes are the most external cellular envelopes, which separate the cells from the surrounding environment and, in the case of eukaryotic cells, compartmentalize them into different internal organelles [1]. Cell membranes exhibit a complex composition including several different species of lipids and proteins. The lipids, in particular, are responsible for providing the membrane structural scaffold. The amphiphilic structure of the majority of the lipids promotes their self-assembling into a bilayer-like structure (Figure 1). The lipid bilayer incorporates transmembrane proteins across the bilayer hydrophobic region and also provides a hydrophilic and charged surface for the transient or permanent anchoring of soluble proteins (i.e., peripheral proteins) or other biomolecules [2]. In addition, specific interactions between membrane proteins and lipids occur within cell membranes, and they are crucial for several biological processes [2,3]. The lipids are also energy and heat sources and can be signaling molecules [4]. Altogether, the synergistic functions of proteins and lipids enable cell membranes to act as selective barriers, and their proper functioning is vital for the cells [5].

Among the different types of mammalian cell membranes, the plasma membrane is often the target of pathogens, e.g., viruses, as well as of selective drugs [2]. The plasma membrane is the most outer cell membrane and is primarily composed of three different classes of lipids: glycerophospholipids, sphingolipids, and sterols [6,7]. As reported in Figure 1, most of the lipids exhibit both a hydrophilic and polar headgroup (outlined in red), together with typically two hydrophobic and apolar acyl chains (outlined in yellow). In the case of the glycerophospholipids, the headgroups show the common structural motif including a glycerol unit bound to the phosphate group. An additional molecular group is also bound to the phosphate, which enables the glycerophospholipids to be classified as: phosphatidylcholine (PC), phosphatidyletholamine (PE), phosphatidylserine (PS), phosphatidylglycerol (PG), phosphatidylinositol (PI), and phosphatidic acid (PA). Sphingolipids lack the glycerol unit; instead, their headgroups include an amino-alcohol bound to the phosphate, to which another molecular group is also attached. As in the case of the glycerophospholipids, the chemical identity of this additional molecular group defines the sphingolipid classes. The most abundant sphingolipids are: ceramides (Cer), sphingomyelin (SM), and gangliosides (GM). Acyl chains with different length and level of unsaturation are bound to glycerophospholipid and sphingolipid headgroups. Therefore, the chemical diversity of the lipids in cell membranes is extremely large and varies considerably depending on the specific organisms and even within the same cell, from one membrane type to another [6,8]. Cholesterol (CHOL) is the only sterol in mammalian cell membranes [7]. It exhibits a considerably different chemical structure compared to the other membrane lipids. Indeed, the hydrophilic region of the cholesterol molecule accounts only for a single hydroxyl group, whereas the rest of the molecule is bulky and hydrophobic (Figure 1b).

The cholesterol content in the mammalian membranes can vary between 20 and 50 mol%, and the plasma membrane is typically quite rich in cholesterol with a concentration in the range of 40–50 mol% [9]. The remaining 60–50 mol% of the lipids in the plasma membrane are composed by glycerophospholipids and sphingolipids. In the erythrocyte plasma membrane, one of the most studied mammalian plasma membranes, approximately 30 mol% of the phospholipids are PC, ∼26 mol% SM, ∼27 mol% PE, and ∼17 mol% anionic phospholipids, i.e., PS, PI, and PA [10]. Concerning the acyl chain composition, acyl chains with 16 and 18 C atoms account for 80 mol% of the total phospholipids of which about 30 mol% exhibit saturated acyl chains, while 43 mol% and 22 mol% are monounsaturated and polyunsaturated, respectively [11].

The composition of the two layers of lipids within the lipid bilayer, i.e., the leaflets, is not identical. In the mammalian plasma membrane, the cytosolic leaflet, facing the inside of the cell, is richer in anionic phospholipids, such as PS and PI [12]. On the other hand, the outer leaflet, facing the extracellular matrix, is richer in PC, SM, and cholesterol [13]. Specific membrane proteins named flippases have the fundamental function of maintaining the different compositions of the inner and outer membrane leaflets [14]. Indeed, the loss of the lipid asymmetry triggers specific biological processes [15]. As an example, the exposure of PS lipids on the cell surface has a central role in the initiation of blood coagulation and cell apoptosis [16].

Altogether, cell membranes are very complex systems, even if only their lipid components are taken into account. Thousands of different lipid species are part of the mammalian cell membranes, and the biological function of this high compositional complexity is still poorly understood [6,8]. Indeed, cell membranes are difficult to extract and manipulate, and their complex composition often prevents their direct characterization through biophysical methods [17]. On the other hand, biomimetic lipid membranes, which contain fewer lipid species than real cellular membranes, can be easily prepared in the lab and are compatible with multiple techniques for their structural and dynamic investigation [18,19]. These simpler lipid membranes can be used as model systems, as some of their physical and chemical properties are most likely similar in real cell membranes. Lipid phase transitions [20,21], the bilayer structure [22], the impact of cholesterol on the structure and dynamics of lipid bilayers [23], and the interactions with proteins [24], peptides [25], and drugs [26] are all examples of the detailed information that has been obtained by studying biomimetic lipid membranes.

This review focuses specifically on the advances in the development of biomimetic lipid membranes for biophysical and physico-chemical studies. The discussed biomimetic lipid membranes are aimed at reproducing specific structural and functional properties of the mammalian plasma membrane. In particular, we provide a description of the most common types of biomimetic lipid membranes in solution and on surfaces (Section 2), as well as recent examples of their application for the investigation of protein-lipid and drug-lipid interactions (Section 3). In the Conclusions, we discuss some promising directions for future developments of biomimetic lipid membranes.

## 2. Design of Biomimetic Lipid Membranes

**Figure 2:**
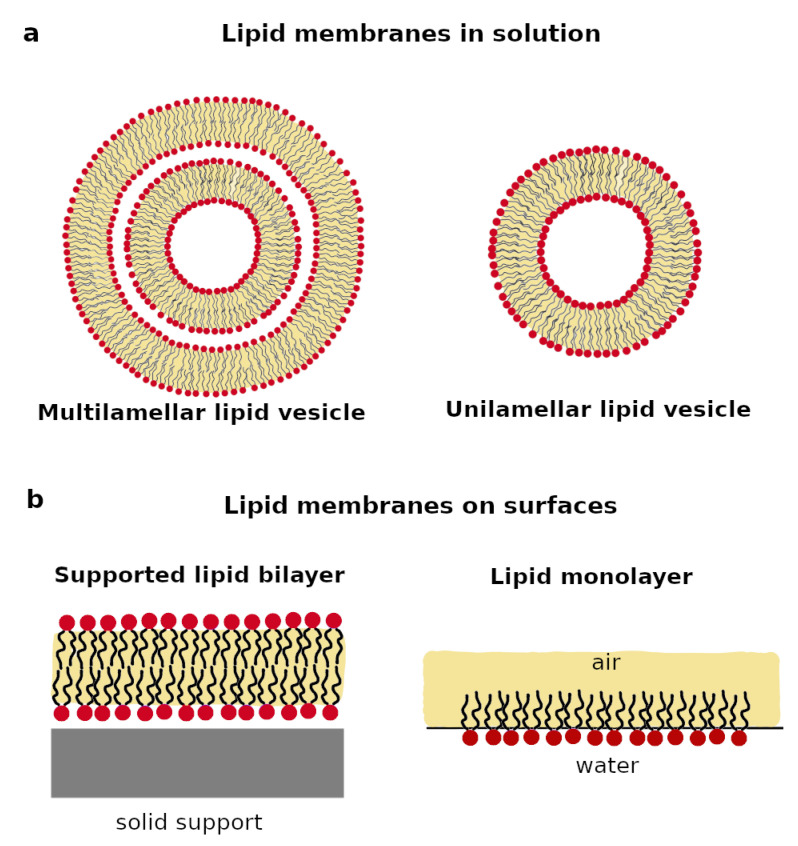
Schematic representation of the most common lipid membranes in solution (**a**) and on surfaces (**b**).

Biomimetic lipid membranes can be divided into two main categories: (1) membranes in solution; (2) membranes on surfaces (Figure 2). These two categories of lipid membranes require different preparation protocols and can be characterized by different biophysical methods. Indeed, scattering techniques, including neutrons, X-ray, and light as probes, spectroscopy techniques, e.g., fluorescence spectroscopy, NMR, electron paramagnetic resonance (EPR) spectroscopy, and calorimetry are typically used for the investigation of lipid membranes in solution [27,28,29]. On the other hand, surface sensitive techniques such as neutron and X-ray reflectometry and diffraction, Langmuir isotherms, attenuated total reflectance-FTIR, and microscopy techniques, e.g., electron microscopy, fluorescence microscopy, atomic force microscopy, and Brewster angle microscopy, are suitable for the characterization of lipid membranes on surfaces [29,30]. In this section, we discuss the main characteristics of the two types of biomimetic lipid membranes together with a brief description of their respective sample preparation protocols.

### 2.1. Lipid Membranes in Solution

Liposomes or vesicles are soluble spherical particles, which are composed of one or more lipid bilayers fused at their ends (Figure 2a). The lipid bilayer is surrounded by water both on the inside and the outside of the vesicle. When the vesicle is composed by a single bilayer, it is named a unilamellar vesicle (UV), whereas a multilamellar vesicle (MV) is composed of several circular bilayers on top of each other, as sketched in Figure 2a. Depending on their sizes, lipid vesicles can be classified as small, i.e., radius < 100 nm, large, i.e., radius > 100 nm, or giant, i.e., radius ≥ 1000 nm [29,31].

Vesicle preparation often starts with dissolving the lipids in an organic solvent and subsequently forming a lipid film by solvent evaporation [32]. The specific choice of the organic solvent strongly depends on the lipid solubility. Chloroform is often used [33,34,35]. However, mixtures of chloroform, methanol, and a small fraction of water were found to be more efficient in solubilizing glycosylated lipids [36,37]. The lipid film is subsequently re-hydrated with water or buffer solutions. Sonication can be applied to improve the solubilization of the lipid film.

Suspensions of small unilamellar vesicles (SUVs) are particularly required for scattering experiments. In order to produce SUVs, the lipid water suspension is typically sonicated or extruded [38,39]. Indeed, when the lipids are initially re-dissolved in water, depending on their composition, they often tend to form multilamellar vesicles with a broad size distribution. Sonication of the vesicle suspension breaks the multilamellar vesicles, which rapidly reassemble into unilamellar vesicles with a more homogeneous size distribution [40]. Extrusion through a polycarbonate membrane also induces temporary rupture of the lipid vesicles and reassembling in unilamellar vesicles [39]. During extrusion, the lipid solution is typically pushed back and forth through the membrane several tens of times, and the cut-off of the membrane defines the final size of the vesicles. However, during this process, a fraction of the lipids, which is difficult to quantify, can remain attached to the membrane, therefore increasing the uncertainty of the final lipid concentration.

Vesicles can be prepared with a large variety of synthetic lipids [41,42]. A recent example is the preparation of vesicles with mixtures of 1,2-dioleoyl-sn-glycero-3-phosphocholine (DOPC) and 1,2-dioleoyl-sn-glycero-3-phospho-L-serine (DOPS), which were used to characterize the binding of Ca${}^{2+}$ ions to the bilayer [43]. Similarly, Poyton and co-workers reported the preparation of vesicles composed of 1-palmitoyl-2-oleoyl-sn-glycero-3-phosphocholine (POPC) and 1-palmitoyl-2-oleoyl-sn-glycero-3-phosphoethanolamine (POPE) for the characterization of Cu${}^{2+}$ binding to these lipids [44]. While Cu${}^{2+}$ is normally present in the cells, an abnormally high concentration of Cu${}^{2+}$ and binding of these ions to the neuronal plasma membrane are correlated to neurodegenerative diseases [44].

As mentioned in the Introduction, the plasma membrane has an asymmetric lipid composition of the inner and outer leaflet. The protocols for vesicle preparation described above typically lead to the production of symmetric vesicles, i.e., inner and outer leaflets of the bilayer exhibit the same lipid composition. However, recently, a sample preparation protocol was developed to produce asymmetric lipid vesicles [45]. In particular, symmetric vesicles are initially formed, and subsequently, the lipids in the outer leaflet are exchanged with different lipid molecules by methyl-β-cyclodextrins. The preparation of small, large, and giant unilamellar vesicles with the outer layer composed of DOPC, SM, and CHOL and the inner leaflet composed of DOPC was recently reported [46]. Such asymmetric vesicles were used to investigate lipid raft domains, which have a fundamental biological function in the mammalian plasma membrane.

Giant unilamellar vesicles (GUVs) can be produced either by natural swelling or electroformation [47]. In the first case, a lipid film is initially formed by drying a lipid solution in an organic solvent. The subsequent slow rehydration of the lipid film leads to the formation of GUVs. The deposition of the lipid film on a polymer support, such as agarose or polyvinyl alcohol (PVA), resulted in improving the GUV formation [48,49]. On the other hand, the production of GUVs by electroformation was originally introduced by Angelova and Dimitrov [50]. It involves the deposition of a lipid film on the surface of two electrodes. The electrodes are immersed in a water solution, and the rehydration of the lipid film, which leads to the GUV formation, is enhanced by the applied electric field [51]. This method has also been adapted to the incorporation of membrane proteins in the GUVs [52].

Lipid mixtures directly extracted from natural sources, such as cells, can also be used to produce lipid membranes, which better resemble the compositional complexity of real cell membranes. In this context, the extraction of plasma membrane vesicles from mammalian cell lines allowed highly realistic models of cell membranes to be investigated [53]. In particular, the isolation of giant vesicles from mammalian cells, which have a very similar lipid composition as the plasma membrane, was used to produce advanced biomimetic membranes for the investigation of lipid rafts or of the mechanical properties of the plasma membrane [54,55,56].

### 2.2. Lipid Membrane on Surfaces

Lipid membranes can be produced in the proximity of both a liquid or a solid surface (Figure 2b). In the case of a liquid surface, a lipid monolayer can be formed at the air–water interface [57,58]. In the monolayer, the lipids are arranged with the headgroups immersed in the water and the acyl chains exposed to air. Lipid monolayers are typically prepared in a Langmuir trough by spreading a lipid organic solvent solution on a water surface, followed by the spontaneous evaporation of the organic solvent. Subsequently, the lipids will spontaneously locate at the air–water interface, therefore producing the monolayer. The surface pressure of the lipid monolayer is regulated by motorized barriers and measured by a Wihelmy’s balance. The collection of Langmuir isotherms on lipid monolayers allows the area occupied by each lipid molecule (i.e., area per lipid) to be directly monitored as a function of the applied surface pressure. Indeed, by properly regulating the surface pressure through the motorized barriers in the Langmuir trough, a compact monolayer with a comparable area per lipid as in cell membranes can be produced [59]. Althougah composed of a single layers of lipids, some dynamic properties of lipid monolayers at the air–water interface, such as lipid layer diffusion, are closer to free-standing cell membranes, whereas lipid membranes on solid support surfaces might be affected by the proximity to the solid support [60].

Lipid monolayers can be prepared with various lipid composition. In general, lipid monolayers are used as membrane biomimics to investigate lipid-phase transitions induced by surface pressure or temperature by collecting Langmuir isotherms [61]. They also allow lipid self-segregation in domains to be directly visualized by Brewster angle microscopy [62]. Finally, the lipid structural arrangement can be studied by reflectivity, both with X-rays and neutrons [58,63].

Recently, monolayers composed by mixtures of 1,2-dipalmitoyl-sn-glycero-3- phosphocholine (DPPC), 1-palmitoyl-2-oleoyl-sn-glycero-3-phospho-(1${}^{\prime }$-rac-glycerol) (POPG), and CHOL were characterized in order to assess the potential biological function of cholesterol as a lipid protector towards chemical degradation [64]. Indeed, compositional analysis by mass spectrometry confirmed the higher tendency of lipid monolayers composed of only phospholipids to be degraded by in situ generated oxidizing agents. Lipid monolayers were also prepared with mixtures of ceramides and sphingomyelin, both belonging to the sphingolipid class, to characterize their rheological properties [65].

When lipid membranes are produced in the proximity of a solid support, the headgroups of the lipids in one leaflet face the support surface, whereas the headgroups of the lipids in the opposite leaflet are exposed to the bulk solvent (Figure 2b). Lipid bilayers can be directly deposited on a hydrophilic support surface, using methods such as vesicle fusion or Langmuir–Blodgett (LB)/Langmuir–Schaefer (LS) deposition [66,67]. During vesicle fusion, a lipid vesicle suspension is injected on a hydrophilic support. Because of the positive interactions between the lipids and the support surface, the vesicles will initially adsorb on the support. The injection of a lower or higher salt solution induces an osmotic shock and promotes vesicle rupture, which leads to the formation of a supported lipid bilayer [68]. On the other hand, the LB/LS method involves the initial formation of a lipid monolayer at the air–water interface, as described above. A solid support with a hydrophilic surface is lifted from the bottom of the trough towards the air–water interface where the monolayer was formed. Lipids are therefore transferred from the air–water interface to the support surface. In a second step, a new lipid monolayer is formed at the air–water interface, and this time, the support with the deposited monolayer is moved from air to water, in order to cross again the air–water interface, but in the opposite direction compared to the first step. This leads to the deposition of a second layer of lipids and the formation of a lipid bilayer. Although vesicle fusion is a simpler method than LB/LS, as it only requires the preparation of a vesicle suspension, it typically produces symmetric lipid bilayers, i.e., the two bilayer leaflets exhibit the same composition [69]. On the other hand, LB/LS can be used to produce asymmetric lipid bilayers, since the two leaflets are deposited in two separate steps and can have a different lipid composition [70].

Lipid bilayers can be also produced on functionalized surfaces. Tethered bilayers can be produced by functionalizing the support surface with an anchor-lipid, i.e., a modified lipid exhibiting a headgroup that can be chemically bound to the support surface [71]. Therefore, the inner leaflet, facing the support, contains a certain fraction of surface-bound lipids, while the rest of the structure is identical to the supported lipid bilayer described above. In order to limit the influence of the support on the physical properties of the lipid bilayer, lipid bilayers can also be produced on supports previously functionalized with a polymer brush or another bilayer, known as a polymer-supported [72] or floating lipid bilayer [73].

Supported lipid bilayers can be produced with a highly variable lipid composition including the most common phospholipids that compose the mammalian plasma membrane [67]. Recently, supported lipid bilayers were used to investigate lipid membranes incorporating glycosylated lipids. Rondelli et al. described different strategies to produce supported lipid bilayers including the ganglioside GM1, one of the most abundant glycosylated sphingolipids in the mammalian plasma membrane [70]. In this study, symmetric supported lipid bilayers were produced by vesicle fusion, while the LB/LS technique was used to produce either supported or floating lipid bilayers incorporating GM1 in the most outer lipid layer. Phosphoinositides (PIPs) are another class of glycosylated lipids that are produced in the mammalian cell membranes by phosphorylation of PI. The phosphorylation can occur at different sites of the inositol ring; however, the most common species in the mammalian plasma membrane are PIP${}_{2}$, PIP${}_{3}$, and PIP${}_{4}$ [74]. Recently, the preparation and characterization of supported lipid bilayers incorporating PIP${}_{3}$ was reported [75]. As a result of the structural characterization, the PIP${}_{3}$ headgroup was shown to be tilted towards the membrane surface and located close to the surrounding PC headgroups. Supported lipid bilayers were also used to investigate the dynamic properties of cell membranes, such as lipid phase transition [76] and lipid flip-flop [77].

The above examples of supported lipid bilayers are based on the application of synthetic lipids or synthetic lipid mixtures. However, natural lipid mixtures can be used as well. In particular, supported lipid bilayers were recently prepared with lipid mixtures extracted from the yeast *Pichia Pastoris* [78,79], which exhibit a composition close to that of the mammalian plasma membrane [80], or lipids extracted from porcine brain tissue [81].

## 3. Applications of Biomimetic Lipid Membranes to Investigate Protein-Lipid or Drug-Lipid Interactions

Lipid-protein interactions play a fundamental role in several biological processes that are vital for the cell. Indeed, proteins can selectively recognize a specific lipid or sense the overall physical properties of cell membranes, e.g., membrane curvature, thickness, or segregated lipid domains [82]. In this context, biomimetic lipid membranes, both in solution [83,84] and on surfaces [85,86], emerged as suitable mammalian plasma membrane models to study protein-lipid interactions through biophysical methods. Indeed, the lipid composition of biomimetic lipid membranes can be suitably tuned to reproduce specific features of cell membranes. This enables the characterization of the impact of specific lipids on membrane-protein interactions [17]. Biomimetic lipid membranes are also largely used as lipid platforms to test drug-membrane interactions, which would occur on the surface of the mammalian plasma membrane. Indeed, more than 60% of the currently marketed drugs target components of the plasma membrane [87].

In this paragraph, we report some relevant examples of biomimetic membranes that were designed to study the interactions with soluble proteins or peptides, as well as other bioactive molecules, such as natural and synthetic drugs. These studies focused on understanding how such interactions affect the biophysical properties of the lipid membranes. They also report the role of specific lipids in driving the mechanisms of action of drugs or in participating at the biological function of proteins and peptides.

### 3.1. Protein-Lipid Interactions

**Figure 3:**
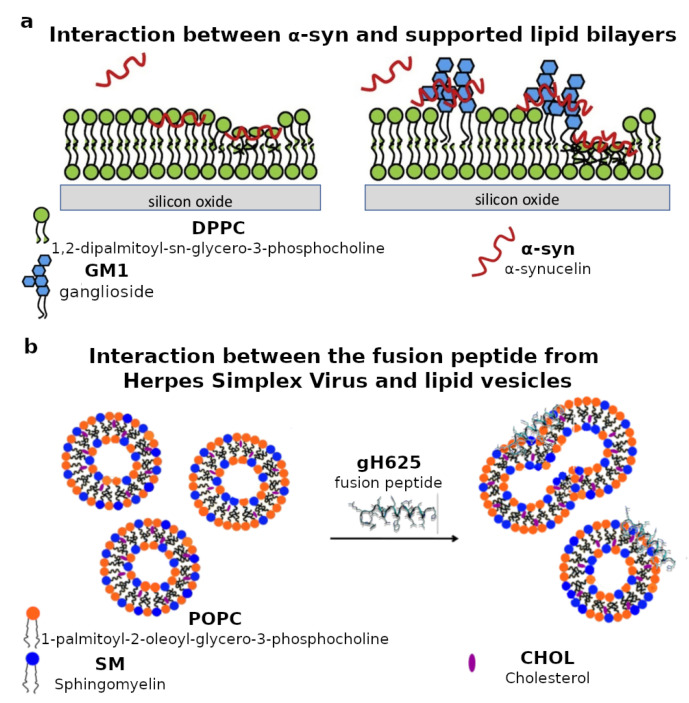
(**a**) Schematic representation of the interaction between α-synuclein (α-syn) and supported lipid bilayers composed of either pure 1,2-dipalmitoyl-sn-glycero-3-phosphocholine (DPPC) or a mixture of DPPC and ganglioside GM1. Neutron reflectometry measurements revealed that GM1 affects the location of α-syn with respect to the biomimetic lipid membrane. Adapted and reprinted with permission from [97], Copyright (2019) Elsevier. (**b**) Schematic representation of the interaction between the gH625 fusion peptide from the envelope of the herpes simplex virus (HSV) and lipid vesicles with lipid composition mimicking the rafts in the mammalian plasma membrane. Electron paramagnetic spectroscopy measurements revealed that the peptide mainly interacts with the lipid headgroups. Adapted and reprinted with permission from [113], Copyright (2015) Royal Society of Chemistry (RSC).

Recently, interactions between lipid membranes and intrinsically disordered proteins have been intensively studied. In particular, the interaction between α-synuclein (α-syn) and biomimetic lipid membranes is one of the most studied because of its implications in Parkinson’s disease, a severe neurological disorder. α-syn is a small soluble protein in its physiological state, but it can aggregate and form fibrils that accumulate in the neurons, therefore causing neurodegenerative pathologies [88]. Many studies investigated the role of lipids in favoring the α-syn aggregation into fibrils [89,90,91]. Indeed, α-syn binding to biomimetic membranes was probed by using both lipid membranes in solution and on surfaces, which were prepared with negatively charged phospholipids, such as PS [92,93] or PG [94]. As a result, the α-syn binding affinity for biomimetic membranes is directly correlated to the amount of negatively charged phospholipids in the membrane. This was recently demonstrated by in vitro experiments investigating the α-syn binding to small unilamellar vesicles, mimicking synaptic vesicles [95]. Monomeric α-syn was shown to interact not only with the lipid headgroups, but also to penetrate the hydrophobic region of supported lipid bilayers prepared with equimolar 1,2-dioleyl-phosphatidyl-glycerol (DOPG) and 1,2-dioleyl-phosphatidyl-ethanolamine (DOPE) [96]. In addition, supported lipid bilayers composed of mixed PC and GM1 ganglioside were recently prepared in order to investigate the selective α-syn/GM1 interaction, which induced strong structural rearrangement of the biomimetic membranes (Figure 3a). This observation is a strong indication of the potential critical role of lipid rafts in the biological function of α-syn [97].

Similarly to α-syn, other amyloidogenic proteins and peptides, with an intrinsically disordered structure and a propensity to self-aggregate into fibrils, also known as amyloids, can interact with biomimetic lipid membranes and are involved in human neurodegenerative diseases. Although the mechanisms of amyloid growth and toxicity are still not fully understood, the interaction between amyloidogenic proteins and lipid membranes is believed to favor protein aggregation into first early oligomers and, subsequently, mature fibrils with specific biophysical, structural, and toxicity features [98]. In particular, the interaction between the amyloid β peptide (Aβ-peptide), which is derived from human islet amyloid polypeptide (IAPP), and biomimetic lipid membranes was recently studied in solution (i.e., giant and large unilamellar vesicles) [99]. The characterization of the interaction between the Aβ-peptide (residues 1–42) and large unilamellar vesicles composed of DOPC revealed the role of the zwitterionic lipid membrane in increasing the fibril growth [100]. In addition, a recent study on a supported lipid bilayer composed of POPC showed that the IAPP can initially absorb on the membrane surface and subsequently perturb the membrane structure by extracting lipids [100]. The presence of membrane domains enriched in cholesterol and sphingolipids was also reproduced in biomimetic lipid membranes and resulted in having a strong impact on the interaction with the Aβ peptide [101]. In this context, the characterization by X-ray diffraction of supported lipid membranes made of dimyristoylphosphatidylcholine (DMPC), dimyristoylphosphoserine (DMPS), and cholesterol (at 30 mol%) demonstrated the effect of the membrane lipid composition in modulating the interactions with Aβ(1–42) and Aβ(25–35) fragments [102]. Additional recent studies on Aβ-membrane interaction used vesicles and supported lipid bilayers with a complex lipid composition, including raft lipids such as cholesterol and sphingomyelin (SM), but also polyunsaturated fatty acids (PUFA) (better known as omega-3 lipids), and showed the central role of omega-3 lipids in favoring a deeper internalization of the peptide among the lipid acyl chains and, consequently, hindering its pathogenic self-aggregation [103,104].

Another example of the interaction between a soluble protein and lipid membranes is the recently reported study of the adsorption of the lipid interaction domain (LID) of the Na${}^{+}$/H${}^{+}$ exchanger isoform 1 (NHE1), a mammalian membrane protein, on supported lipid bilayers composed of POPC and 1-palmitoyl-2-oleoyl-sn-glycero-3-phospho-L-serine (POPS) [105]. In particular, the lipid membrane was shown to have an important role in inducing protein folding at the membrane surface.

Lipid membranes are also used as platforms to investigate the interaction between the mammalian plasma membrane and viral fusion proteins or peptides. Indeed, enveloped viruses are characterized by glycoproteins in their envelopes, which have the function of favoring the fusion between the virus envelope and the mammalian plasma membrane. This membrane fusion event is fundamental for the virus RNA to enter into the host cell [106]. Short fusion peptides derived from viral glycoproteins gp36 of feline immunodeficiency virus (FIV) [107,108] and gp41 of human immunodeficiency virus (HIV) [109] were recently shown to absorb on both lipid vesicles and supported lipid bilayers composed of either POPC or POPC/SM/CHOL mixtures. In particular, the bilayer lipid composition was found to be decisive in favoring the membrane fusion process promoted by these peptides. Lipid vesicles with composition POPC/SM/CHOL were also used to investigate the membrane fusion mechanism induced by the membranotropic fragments of gB and gH glycoproteins of herpes simplex virus (HSV) type I [110,111,112,113]. Altogether, these studies suggested that viral glycoproteins might target raft domains within the mammalian plasma membrane (Figure 3b). In addition, negatively charged phospholipids, such as PS, can also affect the adsorption and the membrane location of fusion peptides, as shown in a recent study involving POPC/POPS vesicles and peptides derived from the C-terminal domain of HIV-1 viral protein R [114]. Glycoproteins of other enveloped viruses such as influenza or SARS-CoV viruses were also shown to have a strong association with raft-like lipid domains [54,115]. Therefore, the plasma membrane lipid composition has a central role in the mechanism and extent of membrane fusion, i.e., the first step of the viral infection [116].

### 3.2. Drug-Lipid Interactions

**Figure 4:**
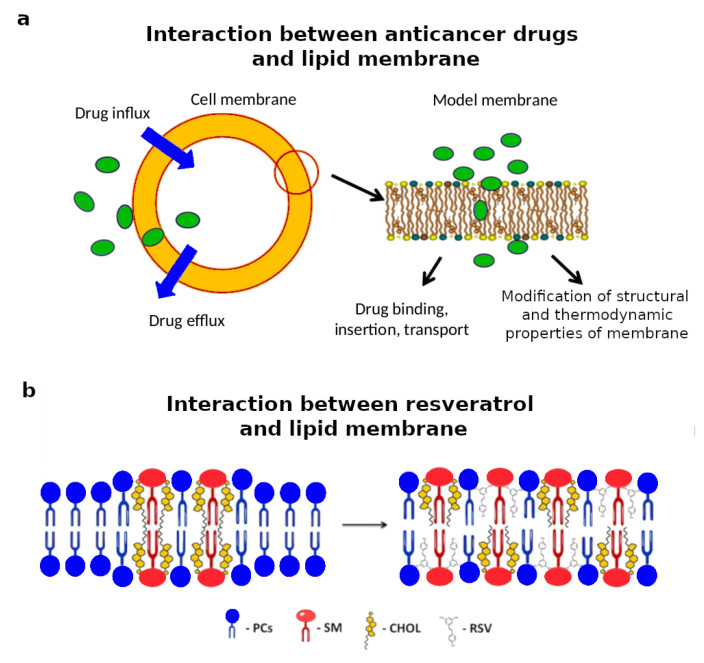
(**a**) Schematic representation of the interaction between anticancer drugs and the cell membrane. Anticancer drugs must cross the plasma cell membrane to reach their internal cellular targets. Adapted and reprinted with permission from [120], Copyright (2014) Elsevier. (**b**) Schematic representation of the interaction of a biomimetic lipid membrane rich in cholesterol and sphingomyelin with resveratrol (RVS), a natural compound with cardioprotective, anti-neurodegenerative, antioxidant, and antitumor action. Adapted and reprinted with permission from [129], Copyright (2016) American Chemical Society (ACS).

The plasma membrane being among the most external cellular components, the characterization of drug-lipid interactions has a central role in the development of new and more efficient drugs of natural or synthetic origins [117]. Among all, the investigation of the interactions between anticancer drugs and lipid membranes is of particular interest because of the peculiar lipidomic profile of cancer cells, which can be potentially used to design selective antitumoral therapies [118]. Lipid monolayers composed of egg yolk phosphatidylcholine lipids with and without cholesterol were used as biomimetic membranes to characterize their interaction with curcumin, a potential anticancer and ant-inflammatory agent [119]. As a result, curcumin molecules had a condensing effect on the lipid monolayer prepared with only phosphatidylcholine lipids, while the opposite effect was observed when cholesterol was also present in the monolayer. Drug-lipid interactions were also reported for chemotherapeutic agents widely used against cancer such as doxorubicin, paclitaxel, cisplatin, gemcitabine, and vinblastine [120]. These drugs target internal components of cancer cells; therefore, understanding the mechanism through which they interact with the plasma membrane lipids and eventually cross the membrane is of great relevance (Figure 4a). In particular, lipid monolayers prepared with either DMPC or mixtures of DMPC and DMPS were used to show doxorubicin’s preferential interaction with negatively charged membranes [121]. Lipid monolayers were also prepared with natural lipid mixtures directly extracted from doxorubicin-sensitive and doxorubicin-resistant cancer cells [122]. This study showed that lipids extracted from doxorubicin-resistant cancer cells exhibit a higher content of sphingomyelin, phosphatidylinositol lipids, and cholesterol compared to doxorubicin-resistant cancer cells. As a consequence of their different lipid composition, the monolayers produced from the resistant cancer cells are more condensed and rigid, and therefore, these cells might reduce the transport of doxorubicin across their plasma membrane.

Anti-inflammatory drugs are another large class of biologically-active compounds. The study of their interaction with the plasma membrane lipids is of great relevance for understanding their mechanism of action. Tomelin, a non-steroidal anti-inflammatory drug for the treatment of rheumatoid arthrosis, was shown to strongly interact with the DPPC lipid headgroup within both biomimetic lipid membranes in solution and on surfaces [123]. The electrostatic interactions between tomelin molecules and the lipid headgroups, which promote tomelin adsorption on the membrane surface, are believed to be the reason for the great drug efficacy, but also for its related side-effects. Evidence of association with the lipid headgroups was also collected for other more common non-steroidal anti-inflammatory drugs (NSAIDs) such as ibuprofen, naproxen, and diclofenac [124]. In particular, in this study, vesicles composed of DMPS were used as biomimetic lipid membranes.

The membrane localization and orientation of ibuprofen, as well as its influence on membrane properties were recently studied by using solid-state NMR spectroscopy and other biophysical assays on large unilamellar vesicles composed of POPC or a POPC/ cholesterol mixture [125]. The experimental results demonstrated that ibuprofen adopted a mean position in the upper chain/glycerol region of the POPC membrane, oriented with its polar carbonyl group towards the aqueous phase. At the same time, the interaction with the membrane was only marginally altered in the presence of cholesterol, in contrast with a previous study indicating that ibuprofen was expelled from the membrane interface in cholesterol-containing DMPC bilayers [126]. Supported lipid bilayers with different composition (i.e., DLPC and POPC, also in mixture with POPG and cholesterol) were chosen to describe the multi-step interactions between lipid membranes and ibuprofen, as a function of its concentration [127]. As a result, both the chemistry of the lipid headgroups and the packing of lipid acyl chains substantially influence the drug-membrane interactions. Unilamellar vesicles composed of DMPC were also used to understand the effect of aspirin, a commonly used NSAID, on the phase behavior of biomimetic membranes [128]. Neutron scattering measurements indicated that aspirin accelerated both lateral and internal motions, with the more pronounced effect observed for the ordered phase of the neat membrane. In particular, aspirin appeared to have a plasticizing effect on the DMPC membrane dynamics, not only on all the measured time scales, but also in both the gel and fluid lipid phase.

The natural polyphenol resveratrol exhibits potential therapeutic activity with cardioprotective, anti-neurodegenerative, antioxidant, and antitumor action. Recently, these therapeutic actions were suggested be related to resveratrol’s interaction with the plasma membrane. In particular, by using biomimetic membranes in solution composed of egg-PC, SM, and cholesterol at different ratios, resveratrol molecules were found to be mainly located in bilayer domains rich in cholesterol and SM; see Figure 4b [129]. In addition, the impact of resveratrol on the phase transition of vesicles composed of DLPC and DSPC was also reported [130]. As a result, resveratrol abolishes the transition of DLPC and acts as a plasticizer for phospholipids with longer fatty acyl chains. The interaction with biomimetic membranes was also investigated for another natural compound, i.e., quercetin, a naturally-occurring flavonoid, which exhibits beneficial health effects [131]. Quercetin showed a strongly pH-dependent tendency to insert into a biomimetic membrane composed of 1,2-diacyl-sn-glycero-3-phosphocholine, which resulted in significant alterations of the membrane’s functioning.

## 4. Conclusions

The mammalian plasma membrane is a very complex system, which is difficult to isolate and directly investigate by conventional biophysical methods [17]. In this context, biomimetic lipid membranes, both in solution and on surfaces, emerged as simpler, but still biologically relevant, model systems to investigate the physico-chemical properties of the mammalian plasma membrane [19]. In this review, we discussed an overview of the most common sample preparation methods to produce biomimetic lipid membranes in solution, i.e., lipid vesicles or liposomes, as well as lipid membranes in the proximity of solid or liquid surfaces, i.e., supported lipid bilayers and lipid monolayers (Section 2). Such biomimetic lipid membranes can be produced with a highly variable lipid composition, therefore allowing for the characterization of the impact of different lipid species on their structure and dynamics. Recent developments in the design of biomimetic lipid membranes involved protocols for producing asymmetric lipid membranes, both in solution [41] and on surfaces [70], and for producing lipid membranes mimicking the rafts in the mammalian plasma membrane [52]. We also discussed some recent applications of the biomimetic lipid membranes for the characterization of their interaction with proteins, as well as drugs (Section 3). In particular, these studies showed the great functional role of lipid rafts in biological interactions at the membrane surface [97,101,113].

Most of the discussed scientific cases focused on the investigation of biomimetic lipid membranes with a relatively simple composition, 1-3 synthetic lipids. The advantage of these lipid membranes is their relatively simple preparation protocols, which allow the concentration of each of the lipid components to be finely tuned. In addition, they are compatible with several biophysical methods, which enable a direct correlation between specific physico-chemical properties of the biomimetic lipid membranes and the presence of a specific lipid component. Evidence of such correlations was reported in the case of supported lipid bilayer prepared with the phosphoinositide PIP${}_{3}$ [75] or the ganglioside GM1 [70]. The lipid composition of the biomimetic lipid membranes can also affect the interactions with proteins and peptides. Nevertheless, these biomimetic lipid membranes lack high compositional complexity, which is one of the main features of the mammalian plasma membrane, where thousands of different lipid species are mixed together [6,7]. We are only recently approaching a deeper understanding of this high compositional complexity, and biophysical studies on lipid membranes can give a great contribution to this research field. Indeed, as discussed in Section 2, several protocols are emerging for producing biomimetic lipid membranes in solution and on surfaces with natural lipid mixtures directly extracted from cells [53,78,79]. Biomimetic lipid membranes composed of natural lipid mixtures represent a considerable advancement in developing more biologically relevant lipid membranes, which can also be implemented for the investigation of membrane interactions with proteins and drugs. Several new insights into the understanding of these interactions can be gained by comparing the previous studies with the simple synthetic lipid membranes and future studies implementing lipid membranes composed of natural lipid extracts.

Besides the implementation of more biologically relevant lipid compositions, future advancements in the development and characterization of lipid membranes as mimics of the mammalian plasma membrane should also include membrane proteins. Indeed, about 50% of the mammalian plasma membrane components are proteins, the structure and function of which strongly depend on the surrounding lipid environment [6]. Recently, sample preparation protocols were proposed for the incorporation of membrane proteins in biomimetic lipid membranes [132,133,134,135,136]. The suggested protocols show great promise for their application in the characterization of mammalian membrane proteins, although most of these studies focused on the investigation of bacterial proteins [132,135]. This is partially due to the more difficult production and purification of mammalian membrane proteins compared to bacterial membrane proteins.

Altogether, increasing the complexity of lipid composition and incorporating membrane proteins are central aspects in the future developments of more advanced biomimetic lipid membranes. The biophysical investigation of such membranes will increase our understanding of the biological functions of the mammalian plasma membrane and will allow us to produce more biologically relevant membrane models as platforms for studying and testing protein-membrane and drug-membrane interactions. As an example, protein-membrane interaction do not only play a fundamental role in our physiological cellular processes, but are also crucial for the infection of cells by pathogens such as viruses. Advanced biomimetic lipid membranes will increase our knowledge about these kinds of interactions and will promote the development of more efficient drugs and vaccines.

## Data Availability

Not applicable.
